# LG-H-PPO: offline hierarchical PPO for robot path planning on a latent graph

**DOI:** 10.3389/frobt.2025.1737238

**Published:** 2026-01-07

**Authors:** Xiang Han

**Affiliations:** China University of Petroleum (East China), Qingdao, China

**Keywords:** latent graph, offline hierarchical PPO, offline reinforcement learning, robot path planning, sparse reward

## Abstract

The path planning capability of autonomous robots in complex environments is crucial for their widespread application in the real world. However, long-term decision-making and sparse reward signals pose significant challenges to traditional reinforcement learning (RL) algorithms. Offline hierarchical reinforcement learning offers an effective approach by decomposing tasks into two stages: high-level subgoal generation and low-level subgoal attainment. Advanced Offline HRL methods, such as Guider and HIQL, typically introduce latent spaces in high-level policies to represent subgoals, thereby handling high-dimensional states and enhancing generalization. However, these approaches require the high-level policy to search and generate sub-objectives within a continuous latent space. This remains a complex and sample-inefficient challenge for policy optimization algorithms—particularly policy gradient-based PPO—often leading to unstable training and slow convergence. To address this core limitation, this paper proposes a novel offline hierarchical PPO framework—LG-H-PPO (Latent Graph-based Hierarchical PPO). The core innovation of LG-H-PPO lies in discretizing the continuous latent space into a structured “latent graph.” By transforming high-level planning from challenging “continuous creation” to simple “discrete selection,” LG-H-PPO substantially reduces the learning difficulty for the high-level policy. Preliminary experiments on standard D4RL offline navigation benchmarks demonstrate that LG-H-PPO achieves significant advantages over advanced baselines like Guider and HIQL in both convergence speed and final task success rates. The main contribution of this paper is introducing graph structures into latent variable HRL planning. This effectively simplifies the action space for high-level policies, enhancing the training efficiency and stability of offline HRL algorithms for long-sequence navigation tasks. It lays the foundation for future offline HRL research combining latent variable representations with explicit graph planning.

## 1 Introduction

With the rapid advancement of robotics, endowing robots with the ability to autonomously navigate in unknown or complex environments has become one of the core challenges in the fields of artificial intelligence and robotics (Martinez-Baselga et al., 2023). Whether for household service robots, warehouse logistics AGVs, or planetary rovers, efficient and safe path planning forms the foundation for accomplishing their tasks. However, real-world navigation tasks often involve long-horizon decision making—where robots must execute a long sequence of actions to reach their destination—while simultaneously facing sparse rewards—clear positive feedback signals are only obtained when the robot ultimately reaches the goal or completes specific subtasks. These two characteristics pose significant challenges to traditional supervised learning and model-based planning methods. Reinforcement learning (RL), particularly deep reinforcement learning (DRL), is considered a powerful tool for addressing such problems due to its ability to learn optimal strategies through trial and error (Barto, 2021).

Standard online RL algorithms, such as Proximal Policy Optimization (PPO) (Schulman et al., 2017), have achieved success in many domains. However, their “learn-while-exploring” paradigm requires extensive interactions with the environment to gather sufficient effective experience. This is often costly, time-consuming, and even hazardous in real robotic systems (Chen et al., 2025).

To overcome the limitations of online RL, offline reinforcement learning (Offline RL) (Levine et al., 2020) emerged. Offline RL aims to learn policies using only pre-collected, fixed datasets, completely avoiding online interactions with the environment. This enables the utilization of large-scale, diverse historical data. However, Offline RL faces its own unique challenge: the out-of-distribution (OOD) action problem. Learned policies may select actions not present in the dataset, and their value estimates are often inaccurate, leading to a sharp decline in performance (Kumar et al., 2020). For long-temporal-horizon and sparse-reward problems, hierarchical reinforcement learning (HRL) offers an effective solution (Kulkarni et al., 2016). HRL decomposes complex tasks into multiple hierarchical sub-tasks. In a typical two-layer architecture, the high-level policy formulates a sequence of subgoals, while the low-level policy executes primitive actions to achieve the current subgoal. This decomposition not only reduces the temporal scale a single policy must handle but also facilitates credit assignment.

In recent years, Offline HRL—the integration of Offline RL and HRL—has emerged as a research hotspot, regarded as a promising direction for tackling complex robotic tasks. Guider (Shin and Kim, 2023) and HIQL (Park et al., 2023) share the common contribution of successfully leveraging latent spaces to handle high-dimensional states (e.g., images) and promote subgoal generalization, while improving sample efficiency through offline learning frameworks. However, they also share a core limitation: high-level policies $\pi_{h}$ must plan and make decisions within a continuous latent space that may be high-dimensional (though lower than the original state space). Concurrently, another research approach attempts to explicitly represent the state connectivity of the environment using graph structures, transforming high-level planning problems into graph search problems. For instance, some online HRL methods (Eysenbach et al., 2019) construct topological maps or empirical maps of the environment during exploration. Recently, GAS (Baek et al., 2025) introduced graph structures into Offline HRL. GAS first learns a Temporal Distance Representation (TDR) space, then constructs a graph within this space based on TDR distances and a Time Efficiency (TE) metric. It employs graph search algorithms (e.g., Dijkstra) to directly select subgoal sequences, replacing explicit high-level policy learning. GAS has achieved significant success on tasks requiring extensive trajectory stitching (Baek et al., 2025). While GAS demonstrates impressive capabilities in trajectory stitching through explicit graph search (e.g., Dijkstra), it relies heavily on the correctness of the constructed graph’s connectivity and the precision of temporal distance estimation. Deterministic search algorithms can be brittle; if the graph contains noisy edges or incorrect connections, the planner may fail to find a feasible path. In contrast, LG-H-PPO learns a stochastic high-level policy on the graph rather than executing a deterministic search. This distinction is crucial:

Robustness: The value function learned by PPO allows the agent to identify and avoid edges that appear feasible in the graph structure but are unreliable for actual traversal, offering greater robustness against imperfect graph construction.

Generalization: A learned policy can better handle states that do not perfectly align with graph nodes, enabling smoother control through probabilistic selection, which is difficult for rigid graph search methods to achieve.

To this end, we propose the LG-H-PPO (Latent Graph-based Hierarchical PPO) framework. Our core idea is to transform the challenging continuous latent variable space from Guider (Shin and Kim, 2023)/HIQL (Park et al., 2023) into a discrete, easily manageable latent variable graph, then enable the high-level PPO to plan on this graph. By simplifying the high-level policy’s action space from a continuous latent variable space to node selection on a discrete latent variable graph, our preliminary experiments on D4RL benchmarks like Antmaze validate our expectations. LG-H-PPO demonstrates significant improvements over Guider (Shin and Kim, 2023) and HIQL (Park et al., 2023) in both convergence speed and final success rate. The main contribution of this paper is the introduction of a new paradigm for offline HRL that combines latent variable representations with explicit graph structures. Theoretically, discretizing the continuous latent space significantly mitigates the complexity of the credit assignment problem in hierarchical policy gradients. In continuous latent space methods (like Guider), the high-level policy must learn a mapping from states to exact latent vectors, where slight deviations in the output can lead to vastly different low-level traversals, causing high variance in gradient estimation. By restricting the high-level policy’s output to a finite set of graph nodes (transforming ‘creation’ into ‘selection’), LG-H-PPO drastically reduces the variance of the policy gradient. This stabilization of the training process allows for more accurate value estimation and significantly improves sample efficiency. By discretizing the high-level action space, we effectively resolve the planning challenges faced by existing methods. This work lays the foundation for future exploration of more efficient and robust graph- and latent variable-based offline HRL algorithms.

## 2 The proposed methods

### 2.1 LG-H-PPO algorithm

**FIGURE 1:**
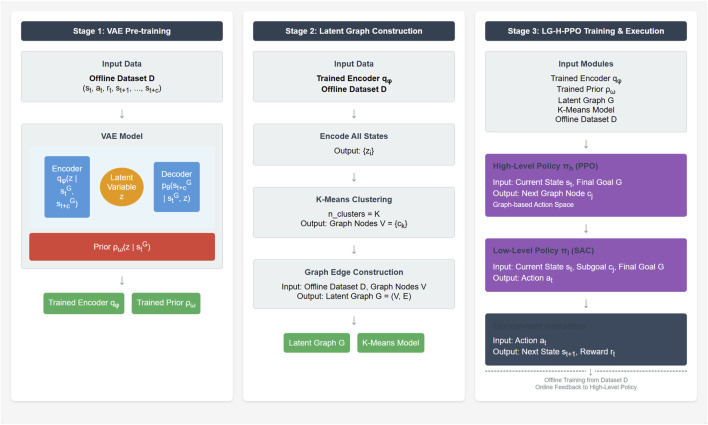
Overall architecture style of LG-H-PPO.

In this section, we present our proposed LG-H-PPO (Latent Graph-based Hierarchical PPO) framework in detail. The core objective of this framework is to significantly reduce the complexity of long-term planning for high-level policies in offline hierarchical reinforcement learning (Offline HRL), particularly within PPO-based frameworks, by introducing a latent variable graph structure. The overall architecture of LG-H-PPO is illustrated in Figure 1, comprising three organically integrated stages: latent variable encoder pre-training, latent variable graph construction, and graph-based hierarchical PPO training.

**FIGURE 2:**
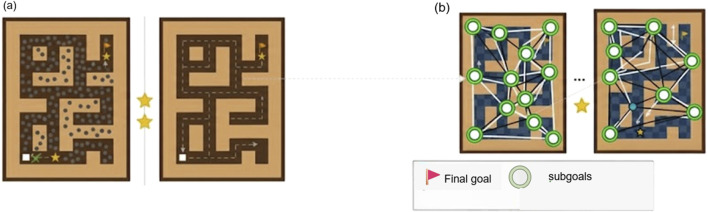
**(a)** The raw Antmaze environment with latent states extracted from the offline dataset. **(b)** The constructed discrete latent graph G=(V,E) , where nodes (subgoals) are formed by clustering latent states, and edges represent learned reachability.

LG-H-PPO follows the fundamental paradigm of HRL by decomposing complex navigation tasks into two levels: high-level (subgoal selection) and low-level (subgoal arrival). The key innovation of LG-H-PPO lies in constructing a discrete latent variable graph G=(V,E) . The task of the high-level policy $\pi_{h}$ is no longer to “create” a latent subgoal z in a high-dimensional continuous space but is simplified to “select” an adjacent node as the next subgoal on this pre-constructed graph G . The low-level policy $\pi_{l}$ executes atomic actions, facilitating transitions between latent variable states represented by graph nodes. The entire framework is designed around training using a fixed offline dataset D to enhance sample efficiency and accommodate scenarios where extensive online interactions are impractical. We apply K-Means clustering to the latent representations z of all states in the offline dataset D to identify K representative cluster centroids, which form the nodes V of our graph. Edges E are then established between nodes that are temporally adjacent in the dataset. This entire process is visualized in Figure 2.

### 2.2 Training process

The first stage involves pretraining a latent variable encoder. The objective is to learn a high-quality, low-dimensional state representation z from offline data, which should capture key state information and reachability relationships between states. We aim to obtain an encoder $q_{\phi }\left(z|s\right)$ . This model primarily consists of three key components: Encoder (Posterior Network) $q_{\phi }\left(z|s_{t}^{G},s_{t+c}^{G}\right)$ : Inputs the current state $s_{t}^{G}$ (mapped to the target space, e.g., coordinates) and the future state $s_{t+c}^{G}$ after c steps, outputting the posterior distribution parameters (mean $\mu_{\mathrm{post}}$ and variance $\sigma_{\mathrm{post}}^{2}$ ) of the latent variable z . Decoder Network $p_{\theta }\left(s_{t+c}^{G}|s_{t}^{G},z\right)$ : Inputs the current state $s_{t}^{G}$ and the latent variable $z∼q_{\phi }$ sampled from the posterior distribution, attempting to reconstruct the state $s_{t+c}^{G}$ after c steps. Prior Network $\rho_{\omega }\left(z|s_{t}^{G}\right)$ : Inputs only the current state $s_{t}^{G}$ and outputs the prior distribution parameters of the latent variable z (mean $\mu_{\mathrm{prior}}$ and variance $\sigma_{\mathrm{prior}}^{2}$ ). All three networks are implemented using Multi-Layer Perceptrons (MLPs) with ReLU or GELU activation functions (Hafner et al., 2020). The training objective maximizes the Evidence Lower Bound (ELBO), combining reconstruction loss and KL divergence regularization terms:

$$L_{\mathrm{V AE}}=E_{z∼q_{\phi }}\left[\log p_{\theta }\left(s_{t+c}^{G}|s_{t}^{G},z\right)\right]-\beta D_{KL}\left(q_{\phi }\left(z|s_{t}^{G},s_{t+c}^{G}\right)\|\rho_{\omega }\left(z|s_{t}^{G}\right)\right)$$

Next comes the core innovation: we constructed a latent variable graph, discretizing the high-dimensional, continuous latent variable space Z learned in Phase 1 into a structured directed graph G=(V,E) . Nodes V represent representative states (or regions) within the latent space, while edges E denote the latent reachability between these states (based on observations in the offline dataset). We use the trained encoder $q_{\phi }$ to encode all states ${\left\{s_{i}\right\}}_{i=1}^{N}$ in the offline dataset D , yielding the corresponding latent variable set $Z={\left\{z_{i}\right\}}_{i=1}^{N}\subset R^{d_{z}}$ , where $d_{z}$ is the latent variable dimension. Select an appropriate number of nodes K (a key hyperparameter, e.g., K = 100, 200, 500). Apply the K-Means algorithm to cluster the latent variable set Z , aiming to minimize the sum of squared errors within clusters:

$$\underset{C}{\text{argmin}\;}\overset{K}{\underset{k=1}{\sum }}\underset{z_{i}\in C_{k}}{\sum }\|z_{i}-c_{k}{\|}^{2}$$

The K cluster centroids $V=\left\{c_{1},\ldots ,c_{K}\right\}$ are defined as the K nodes of the latent variable graph G . Each node $v_{k}$ represents an abstract region within the latent space.

To reflect dynamic reachability between states, we utilize trajectory information from the dataset D to construct graph edges. We traverse each trajectory segment $\tau =\left(s_{t},a_{t},s_{t+1},\ldots ,s_{t+k}\right)$ in D (where k can be a fixed number of steps, such as $c\;H_{TD}$ (Baek et al., 2025), or the entire trajectory). Let $z_{t}=q_{\phi }\left(s_{t}\right)$ and $z_{t+k}=q_{\phi }\left(s_{t+k}\right)$ denote the initial and final latent variables of the segment, respectively. Use the K-Means model to find their nearest node indices: $i={\mathrm{arg min}}_{l}\|z_{t}-c_{l}{\|}^{2}$ and $j={\mathrm{arg min}}_{l}\|z_{t+k}-c_{l}{\|}^{2}$ . If i≠j , this indicates the presence of observations in the dataset moving from node $v_{i}$ ’s region to node $v_{j}$ ’s region. We then add a directed edge $e\left(v_{i},v_{j}\right)\in E$ to the graph G .

$$\begin{array}{ll} & E=\left\{e\left(v_{i},v_{j}\right)|\exists \tau =\left(s_{t},\ldots ,s_{t+k}\right)\in D,i={\mathrm{arg min}}_{l}\|q_{\phi }\left(s_{t}\right)-c_{l}{\|}^{2},j\right.\\ & \;\left.=,{\mathrm{arg min}}_{l},\|,q_{\phi },\left(s_{t+k}\right),-,c_{l},{\|}^{2},,,i,\neq ,j\right\} \end{array}$$

Next, train the hierarchical PPO policy $\pi =\left(\pi_{h},\pi_{l}\right)$ on the constructed latent variable graph G to enable it to effectively utilize the graph structure for long-term planning and navigation. The task of the lower-level policy $\left(\pi_{l}\right)$ is to learn $\pi_{l}\left(a_{t}|s_{t},c_{j}\right)$ , enabling the agent to drive itself from the current state $s_{t}$ to the latent variable region represented by the target node $c_{j}$ . We adopt an offline Soft Actor-Critic (SAC) (Haarnoja et al., 2018) training paradigm based on Advantage-Weighted Action Cloning (AWAC/AWR) (Peng et al., 2019). At the high-level policy level ( $\pi_{h}$ - PPO), our task is to learn $\pi_{h}\left(c_{j}|v_{i},z_{\mathrm{final}}\right)$ , which selects the optimal next neighbor node $c_{j}$ as a sub-goal on the graph G based on the current graph node $v_{i}$ and the final objective (encoded as $z_{\mathrm{final}}$ ). The high-level state consists of the current encoded and mapped graph node $v_{i}$ (or its latent variable $c_{i}$ ) and the latent variable of the final goal $z_{\mathrm{final}}$ . The discrete action space $A_{h}\left(v_{i}\right)=\left\{v_{j}|e\left(v_{i},v_{j}\right)\in E\right\}$ represents the set of all neighboring nodes reached via outgoing edges from a node $v_{i}$ in graph G . During PPO training, the network architecture $\pi_{h}$ comprises Actor and Critic networks, both implemented using MLP. Compute the K logits output by the actor. Simultaneously, a mask is constructed to set the logits of all non-neighboring nodes $v_{k}\notin A_{h}\left(v_{i}\right)$ to −∞ . Subsequently, a Softmax function is applied to the logits of the remaining neighboring nodes to obtain a probability distribution, from which the next node is sampled: $c_{j}∼\pi_{h}\left(\cdot |v_{i},z_{\mathrm{final}}\right)$ . The high-level PPO relies on round data $\left(v_{t},c_{t+1},R_{h,t},v_{t+1}\right)$ , which we collect through simulated interactions. Using the collected high-level rollout data, we compute the Generalized Advantage Estimate (GAE) (Schulman et al., 2015):

$${\hat{A}}_{t}=\overset{N-1}{\underset{l=0}{\sum }}{\left(\gamma \lambda \right)}^{l}\delta_{t+l},\;\text{where }\delta_{t}=R_{h,t}+\gamma V_{\phi }\left(v_{t+1},z_{\mathrm{final}}\right)-V_{\phi }\left(v_{t},z_{\mathrm{final}}\right)$$

Specifically, to stabilize the high-level policy updates and prevent catastrophic policy collapse, we employ the standard clipped surrogate objective function of PPO. The optimization objective $L^{\mathrm{CLIP}}\left(\theta \right)$ for the high-level policy network $\pi_{\theta }$ is defined as:

$$L^{\mathrm{CLIP}}\left(\theta \right)={\hat{E}}_{t}\left[\min\left(r_{t}\left(\theta \right){\hat{A}}_{t},\text{clip}\left(r_{t}\left(\theta \right),1-\epsilon ,1+\epsilon \right){\hat{A}}_{t}\right)\right]$$

where $r_{t}\left(\theta \right)=\frac{\pi_{\theta }\left(c_{j}|v_{i},z_{\mathrm{final}}\right)}{\pi_{\theta_{\mathrm{old}}}\left(c_{j}|v_{i},z_{\mathrm{final}}\right)}$ denotes the probability ratio between the new and old policies, and ${\hat{A}}_{t}$ is the Generalized Advantage Estimate (GAE) computed via Equation 3. ϵ is the clipping hyperparameter (set to 0.2 in our experiments). This objective ensures that the updated policy does not deviate excessively from the behavior policy that generated the data, guaranteeing monotonic improvement during training. Subsequently, we update the Actor and Critic networks using multiple iterations (epochs) and mini-batches of data, minimizing the joint loss function of PPO. This ultimately yields the trained low-level policy $\pi_{l}$ and high-level PPO policy $\pi_{h}$ .

## 3 Experiment and result analysis

This section aims to evaluate the effectiveness of our proposed LG-H-PPO framework through a series of rigorous experiments. We compare LG-H-PPO with current offline hierarchical and non-hierarchical reinforcement learning algorithms on the challenging D4RL Antmaze navigation benchmark. The experimental design focuses on validating LG-H-PPO’s performance advantages in addressing long-temporal-order, sparse-reward problems, particularly in convergence speed, final performance, and training stability. Furthermore, we conduct ablation studies and qualitative analyses to delve into the critical role of latent variable graph structures and the internal workings of the framework.

### 3.1 Experimental design

We evaluate on the Antmaze navigation benchmark, focusing on antmaze-medium-diverse-v2 and antmaze-large-diverse-v2. These environments simulate navigation tasks for quadrupedal robots in medium and large mazes, characterized by high state space dimensions (29-dimensional), continuous action space (8-dimensional), limited field of view, sparse rewards (+1 only upon reaching the goal), and long task durations (up to 1,000 steps). They serve as an ideal platform for testing long-term planning and offline learning capabilities. Training is based on the antmaze-medium-diverse-v2 and antmaze-large-diverse-v2 offline datasets. The diverse dataset contains a large number of suboptimal trajectories generated by medium-level policy exploration, offering broad coverage but few successful trajectories. This places high demands on the algorithm’s trajectory stitching capabilities and its ability to learn optimal policies from suboptimal data (Baek et al., 2025; Shin and Kim, 2023). Each dataset contains approximately one million transition samples.

Baselines: To comprehensively evaluate LG-H-PPO’s performance, we selected the following representative baselines for comparison:

1. Guider (Shin and Kim, 2023): A state-of-the-art offline HRL algorithm based on VAE latent variables and continuous high-level actions. It serves as a crucial foundation and benchmark for our approach.
2. HIQL (Park et al., 2023): A state-of-the-art offline HRL algorithm based on implicit Q-learning and latent variable states as high-level actions. It represents another value-learning-based technical approach to HRL.
3. GAS (Baek et al., 2025): The latest state-of-the-art offline HRL algorithm based on graph structures and graph search, which does not learn explicit high-level policies and excels at trajectory splicing.
4. CQL + HER (Kumar et al., 2020): A state-of-the-art non-hierarchical offline RL algorithm, combined with Hindsight Experience Replay (Andrychowicz et al., 2017) to handle sparse rewards, and used to demonstrate the advantages of hierarchical structures.
5. H-PPO (Continuous): Our baseline implementation adopts the same PPO algorithmic framework as LG-H-PPO, but its high-level policy directly generates actions in the continuous latent variable z space. This serves as the most direct ablation subject for LG-H-PPO, used to validate the improvements brought by discrete graph structures.

We use normalized scores as the primary performance metric. This score is linearly scaled based on the environment’s raw rewards, where 0 corresponds to the performance of a random policy and 100 corresponds to the performance of an expert policy. We run each algorithm and environment with five different random seeds, reporting the final policy’s average normalized score, standard deviation, maximum, and minimum over 100 evaluation rounds. Additionally, we plot the average normalized score curve (learning curve) during training to compare the convergence speed and training stability of different algorithms. We implement the LG-H-PPO framework using PyTorch. Clustering Implementation Details: For the latent graph construction, we utilize the KMeans module from the Scikit-learn library. To ensure high-quality cluster center initialization and faster convergence, we explicitly employ the ‘k-means++’ initialization strategy rather than random initialization. This is critical for generating representative graph nodes in the complex high-dimensional latent space. Discussion on Node Count K: The number of graph nodes K is a key hyperparameter balancing ‘planning resolution’ and ‘computational complexity’. A smaller K may result in nodes representing overly large regions, losing local details, while an excessively large K significantly increases the action space for the high-level policy, complicating learning. In our experiments, K=200 was empirically selected as the optimal value for the Antmaze tasks. Detailed hyperparameter settings are shown in Table 1. All experiments are conducted on a server equipped with an NVIDIA RTX 4090 GPU.

**TABLE 1:** LG-H-PPO hyperparameters.

Component	Hyperparameter	Value
VAE	Network (encoder)	MLP (512, 512, 512)
	Network (decoder)	MLP (512, 512, 512)
	Network (prior)	MLP (512, 512, 512)
	Latent dimension $d_{z}$	16
	KL weight β	0.1
	Subgoal period c	25
	Learning rate	$3\times 10^{-4}$
	Batch size	100
Graph construction	Number of nodes K	200
	Clustering algorithm	K-means (scikit-learn, k-means++)
Low-level $\pi_{l}$	Policy type	SAC + AWAC
	Network (actor)	MLP (256, 256)
	Network (critic)	MLP (256, 256)
	Learning rate	$3\times 10^{-4}$
	AWAC temperature λ	1.0
	Batch size	512
	Discount factor $\gamma_{l}$	0.98
High-level $\pi_{h}$	Policy type	PPO
	Network (actor)	MLP (256, 256, 256)
	Network (critic)	MLP (256, 256, 256)
		
	Discount factor $\gamma_{h}$	0.99
	GAE λ	0.95
	Clip ϵ	0.2
	VF coefficient $c_{1}$	0.5
	Entropy coefficient $c_{2}$	0.01
	PPO epochs	10
	Minibatch size	64
	Learning rate	$3\times 10^{-4}$
	Rollout length (high-level steps)	2048
	High-level decision frequency	c=25

### 3.2 Results and analysis

We summarize the final performance of LG-H-PPO and various baseline algorithms on the D4RL Antmaze task in Table 2. To present the results more comprehensively, we report the average normalized score, standard deviation, and maximum and minimum scores across 100 evaluation rounds for five random seeds.

**TABLE 2:** Performance comparison on D4RL antmaze tasks.

Environment	Metric	LG-H-PPO (Ours)	Guider (Martinez-Baselga et al., 2023)	HIQL (Barto, 2021)	GAS (Baek et al., 2025)	CQL + HER (Shin and Kim, 2023)	H-PPO (Cont.)
Antmaze-medium-diverse-v2	Mean score	90.5	87.3	89.9	96.3	28.3	75.2
	Std. Dev	± 3.1	± 0.4	± 4.5	± 1.3	± 5.3	± 4.5
	Max	94.8	88.1	95.0	98.0	35.5	81.0
	Min	85.2	86.5	85.0	94.8	19.8	69.5
Antmaze-large-diverse-v2	Mean score	85.6	80.8	88.2	93.2	11.3	68.1
	Std. Dev	± 4.2	± 4.6	± 3.0	± 0.8	± 8.2	± 5.8
	Max	91.5	87.0	92.0	94.5	22.0	75.3
	Min	79.8	75.1	84.0	92.1	0.5	60.2

Table 2 clearly demonstrates the superiority of LG-H-PPO. In the antmaze-medium environment, the average scores of all hierarchical methods significantly outperform the non-hierarchical CQL + HER, highlighting the inherent advantage of hierarchical structures in handling long-temporal sequence problems. LG-H-PPO achieves a high score of 90.5 on this task, matching the performance of top methods HIQL and GAS while significantly outperforming continuous latent space planning-based approaches like Guider and H-PPO (Cont.). This performance advantage is further amplified in the more challenging antmaze-large environment. Confronted with longer paths and sparser rewards, the non-hierarchical method CQL + HER experiences a steep decline to 11.3. Guider and H-PPO (Cont.) also drop to 80.8 and 68.1 respectively, indicating significantly increased difficulty in long-term planning within continuous latent spaces. In contrast, LG-H-PPO, leveraging its planning capability on discrete latent variable graphs, maintained a high performance of 85.6. This substantially outperformed both Guider and H-PPO (Cont.), coming very close to HIQL’s performance. This strongly demonstrates that latent variable graph structures are key to overcoming the bottlenecks of long-term offline HRL planning. By discretizing the action space of high-level PPO, we enable policy gradient methods to more effectively learn long-range dependencies and select optimal subgoal sequences. Concurrently, LG-H-PPO exhibits a relatively small standard deviation, and the gap between its maximum and minimum values indicates stable performance across different random seeds.

**FIGURE 3:**
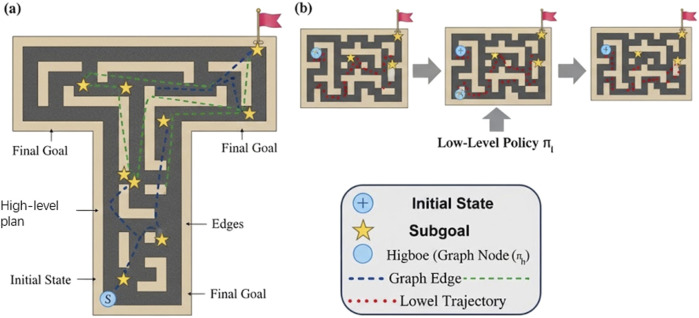
Visualization of LG-H-PPO’s hierarchical planning and execution. **(a)** The high-level PPO policy selects a sequence of discrete graph nodes (yellow stars) as subgoals. **(b)** The low-level policy executes trajectories (red dotted line) to reach each sequential subgoal, successfully navigating from the initial state to the final goal.

To provide a more intuitive understanding of LG-H-PPO’s decision-making process, we visualize a planned trajectory in the Antmaze-Large environment in Figure 3. The visualization highlights the two-level hierarchical structure.As shown in Figure 3a, the high-level PPO policy $\left(\pi_{h}\right)$ , operating on its discrete action space (the graph nodes), selects an efficient sequence of latent subgoals (yellow stars) from the start state (S) to the final goal. This demonstrates its long-term planning capability. Figure 3b shows the low-level policy $\left(\pi_{l}\right)$ in action, executing primitive actions to successfully navigate and reach each of the discrete subgoals provided by the high-level.This qualitative result visually confirms that our framework effectively decomposes the complex, long-horizon task into a series of simpler, short-horizon navigation problems, validating the efficacy of our latent graph-based approach.

## 4 Conclusion and future work

The main contribution of this paper is the proposal and validation of a novel offline HRL paradigm (LG-H-PPO) that integrates latent variable representation learning with explicit graph structure planning. By discretizing the action space of high-level PPO, we effectively overcome the bottlenecks of existing offline HRL methods based on policy gradients, which suffer from low planning efficiency and poor stability in continuous latent spaces. This work lays a solid foundation for future exploration of more efficient and robust offline HRL algorithms that integrate the abstractive capabilities of latent variables with the advantages of explicit structured planning, particularly in robotic applications requiring long-term reasoning and suboptimal data utilization.

Future research directions hold great promise. First, exploring the learning of edge weights in the graph—such as adopting time efficiency metrics from GAS (Baek et al., 2025) or directly learning edge reachability probabilities/transition costs—and integrating this information into the decision-making process of high-level PPO or as reward shaping signals for low-level policies could enable smarter path selection. Second, online dynamic graph expansion mechanisms can be investigated, allowing agents to dynamically add or modify graph nodes and edges based on new experiences during (limited) online interactions or deployment. This enables the discovery of optimal paths potentially missing in offline data, endowing the algorithm with lifelong learning capabilities. Finally, extending the LG-H-PPO framework to navigation tasks based on high-dimensional observations (e.g., images) represents a significant direction. This requires investigating more robust visual encoders and exploring how to effectively construct and utilize graph structures within visual latent spaces.

## Data Availability

The original contributions presented in the study are included in the article/supplementary material, further inquiries can be directed to the corresponding author.
